# Caregiving Experiences of Caregivers of Adolescents With Inflammatory Bowel Disease: A Qualitative Meta‐Synthesis

**DOI:** 10.1002/nop2.70267

**Published:** 2025-06-27

**Authors:** Huan Xu, Chao Wu, Changchang Chen, Bo Yan, Ning Zha, Kai Zhang, Fang Liu, Hongjuan Lang

**Affiliations:** ^1^ School of Nursing Shaanxi University of Chinese Medicine Xianyang China; ^2^ Department of Nursing Air Force Medical University School of Nursing Xi'an China

**Keywords:** adolescent, caregiver, inflammatory bowel disease, meta‐synthesis, qualitative research

## Abstract

**Background:**

Inflammatory Bowel Disease (IBD) is a chronic condition that significantly affects the physical and mental health of adolescents. Caregivers are crucial in the treatment process, offering both physical care and emotional support. Caring for adolescents with IBD can significantly affect caregivers' physical, psychological and daily lives, presenting a range of emotional, financial and practical challenges.

**Aims:**

To explore and integrate the caregiving experiences of caregivers of adolescents with IBD, in order to inform future caregiving support and intervention strategies.

**Design:**

A systematic evaluation and synthesis of qualitative studies was conducted based on ENTREQ guidelines.

**Methods:**

A systematic search was performed in PubMed, Embase, Cochrane Library, Web of Science, CINAHL, PsyINFO, CNKI, Wangfang and VIP for qualitative studies on the caregiving experiences of caregivers of adolescents with IBD, from the start of the research until March 14 2025. The quality of the literature was assessed using the Joanna Briggs Institute's quality assessment criteria for qualitative research, and included studies were integrated using a pooled approach.

**Results:**

In total, nine studies were included, from which 69 themes were extracted. The findings were grouped into five main themes and 12 subthemes: Psychological and emotional challenges; Heavy caregiving burden; Multiple challenges; Indispensable external support; Positive progress.

**Conclusions:**

Caregivers of adolescents with IBD face numerous challenges. Healthcare professionals should recognise the varied needs of these caregivers and develop tailored support programmes to help alleviate psychological stress and enhance caregiving quality. Additionally, the Internet and community platforms can be utilised to establish a family support system, ensuring the well‐being of both caregivers and adolescents.

**No Patient or Public Contribution:**

This study is a meta‐synthesis and does not require relevant contributions from patients or the public.

**Trial Registration:**

PROSPERO number: CRD 420250654435

## 1 Introduction

Inflammatory bowel disease (IBD) is a chronic, idiopathic condition of the intestines, mainly encompassing Crohn's Disease (CD) and Ulcerative Colitis (UC) (Mozdiak et al. 2015). Recent data indicate a significant increase in the incidence and prevalence of IBD in adolescents, both in Europe and North America, with similar trends observed in Asia and South America. In Northern France, the number of adolescents aged 6 to 16 living with IBD tripled between 1988 and 2017. Similarly, in the UK, the number of adolescents living with IBD has doubled over the past 20 years (Ashton and Beattie 2024; Kuenzig et al. 2022; Sarter et al. 2024). Studies have shown that adolescent IBD cases are more complex and severe than those in adults, with a risk of developing malignant complications, including colorectal cancer and toxic megacolon (Olén et al. 2019; Oliveira and Monteiro 2017). Adolescence is a critical period of physical and mental development. Recurrent episodes, complex treatments and the prolonged duration of IBD can negatively impact both physical and psychological health. This includes growth retardation, delayed puberty and severe psychological distress. Adolescents with IBD have a higher prevalence of anxiety and depression compared to those with other chronic conditions (Amaro and Chiarelli 2020; Rosen et al. 2015).

Caregivers play a crucial role in the management of persons living with IBD, taking on both physical care and providing essential psychological and emotional support. Studies have shown that caregiving for persons living with chronic diseases is vital not only for providing emotional and quality of life support, but also for the physical, mental and emotional well‐being, as well as the quality of life, of caregivers (Qiu et al. 2019; Shukla et al. 2018). Adolescents typically lack self‐care skills, so most caregiving tasks fall to caregivers. Adolescent caregivers face a greater burden than those caring for adults. Several studies highlight that caregivers of adolescents with IBD face numerous challenges. Daily caregiving tasks negatively affect their physical and mental health, as well as their social functioning. The high costs of treatment and lost productivity are particularly burdensome (El‐Matary et al. 2022; Kahn et al. 2017; Velasco Rodríguez‐Belvís et al. 2024).

In recent years, there has been growing attention to the caregiving experiences and needs of adolescent caregivers of persons living with IBD, both domestically and internationally, with an increasing number of related qualitative studies. However, a single study cannot fully capture these experiences in an accurate and comprehensive manner. This study applied meta‐synthesis to explore the caregiving experiences of adolescent caregivers of persons living with IBD, aiming to provide theoretical support for policy development, caregiver training and interventions in related fields. This systematic analysis aims to provide a scientific basis for future improvements in the caregiving system for persons living with IBD, as well as to enhance the quality of life for both adolescents living with IBD and their families.

## 2 Methods

### 2.1 Study Design

This study employed a qualitative meta synthesis approach to systematically integrate and analyse existing qualitative research on caregiving experiences of caregivers of adolescents with IBD. The study adhered to the guidelines of the enhancing transparency in reporting of qualitative research syntheses (ENTREQ) statement (Data S1).

### 2.2 Search Strategy

**FIGURE 1:**
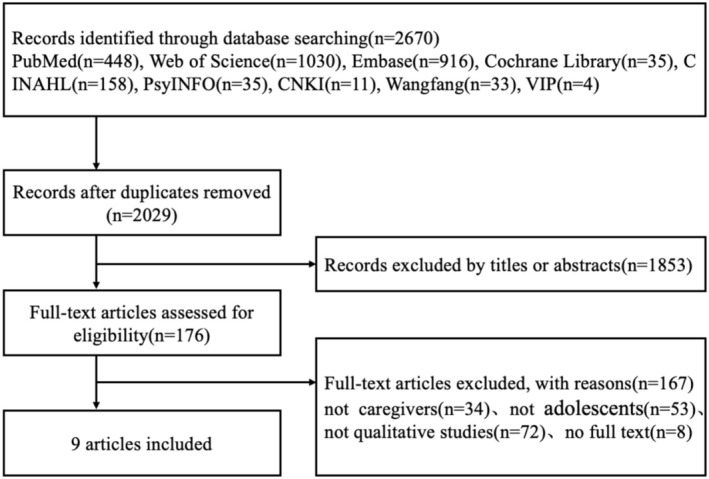
Literature screening process.

A systematic search was conducted across multiple databases, including PubMed, Embase, Cochrane Library, Web of Science, CINAHL, PsyINFO, CNKI, Wangfang and VIP. The search timeframe extended from the time of building the database to March 14, 2025, focusing on qualitative research exploring caregiving experiences of caregivers of adolescents with IBD. The search strategy employed a combination of subject headings (MeSH) and free words, using search terms such as ‘adolescent/teen*/youth*/child*/juvenile*’ ‘parents/family members/care*/relative*/guardian*’ ‘inflammatory bowel diseases/colitis, ulcerative/crohn disease/ulcerative colitis’ ‘qualitative research/grounded theory/focus groups/qualitative study/phenomenon/ethnography/narrative/thematic analysis/experience*/feeling*/interview*’. The full search strategy is in Figure 1 and Data S2.

### 2.3 Inclusion and/or Exclusion Criteria

The study applied the PICO(S) framework to develop inclusion criteria: Population (P): Caregivers (including parents, relatives or other primary caregivers) of adolescents with IBD; Phenomenon of Interest (I): experiences, feelings and challenges during the caregiving process; Context (Co): Long‐term care settings in family or healthcare institutions; Study Design (S): qualitative or mixed studies (only the qualitative part was extracted), including phenomenological research, grounded theory, thematic analysis, ethnographic research, narrative research and focus groups. Exclusion criteria: (1) inaccessible full text; (2) duplicate or incomplete content; and (3) non‐English literature.

### 2.4 Study Selection and Data Extraction

Literature screening and data extraction were conducted independently by two researchers, with cross‐checking upon completion. We identified 2670 studies through searching and used EndNote 21 software to screen the literature. After removing 641 duplicates, we excluded 1853 irrelevant studies based on title and abstract review, and excluded an additional 167 studies after full‐text review, ultimately including nine studies. In case of disagreement between the two researchers, a third researcher was consulted, and a consensus was reached. Data extraction included the authors, publication year, country, study population, methodology, study content and main results.

### 2.5 Quality Appraisal

Two researchers, both trained in evidence‐based nursing, evaluated the quality of the literature using the Joanna Briggs Institute (JBI) quality assessment criteria for qualitative research (Porritt et al. 2014). Ten items were evaluated, each judged as ‘yes’, ‘no’, or ‘unclear’. Level A literature met all criteria and had the lowest likelihood of bias; Level B literature met some criteria and had a moderate likelihood of bias; Level C literature did not meet any criteria and had the highest likelihood of bias. In the event of disagreement between the two researchers, the results were discussed and finalised with a third researcher. Only Level A and B literature were included in this study, while Level C literature was excluded (Table 1).

**TABLE 1:** Quality appraisal.

Author, year	①	②	③	④	⑤	⑥	⑦	⑧	⑨	⑩	Level
Bihari, 2025	Y	Y	Y	Y	Y	N	N	Y	Y	Y	B
Li, 2024	Y	Y	Y	Y	Y	N	Y	Y	Y	Y	B
Zhou, 2023	Y	Y	Y	Y	Y	Y	Y	Y	Y	Y	A
Zhang, 2022	U	Y	Y	Y	Y	N	N	Y	Y	Y	B
Gorbounova, 2022	Y	Y	Y	Y	Y	N	N	Y	N	Y	B
Easterlin, 2020	Y	Y	Y	Y	Y	N	N	Y	Y	Y	B
Kluthe, 2018	Y	Y	Y	Y	Y	N	N	Y	Y	Y	B
Gray, 2015	Y	Y	Y	Y	Y	N	Y	Y	Y	Y	B
Hommel, 2011	Y	Y	Y	Y	Y	N	Y	Y	Y	Y	B

*Note:* Domains: ① Consistency between the stated philosophical perspective and the research methodology; ② Consistency between the research methodology and the research question or objectives; ③ Consistency between the research methodology and the methods used to collect data; ④ Consistency between the research methodology and the representation and analysis of data; ⑤ Consistency between the research methodology and the interpretation of results; ⑥ Locating the researcher culturally or theoretically; ⑦ Addressing the influence of the researcher on the research, and vice versa; ⑧ Representation of participants and their perspectives; ⑨ Ethical approval obtained from an appropriate body; and ⑩ Relationship between conclusions and the analysis or interpretation of data.

Abbreviations: N, No; U, Unclear; Y, Yes.

### 2.6 Data Synthesis

This study integrated the selected literature using the ‘Pooled Integration Approach to Meta‐Synthesis’ (Lockwood et al. 2015), as recommended by the JBI Center for Evidence‐Based Health Care. Specifically, the following steps were undertaken: (1) extraction of key findings and relevant data from the original study to ensure the completeness and accuracy of the information; (2) repeated reading of the data by two independent researchers, open coding using NVivo 14, and identification of core concepts and ideas; (3) integration of similar or related codes to distill higher‐level themes and construct a thematic framework; and (4) multiple reviews of the theme definitions and their internal relationships, followed by negotiation and resolution of disagreements in coding and theme categorization to ensure consistency and reliability.

## 3 Results

A total of 2670 documents were identified through the established search strategy. After rigorous screening and quality assessment by the research team, nine documents that met the inclusion criteria were selected. 140 caregivers participated in these studies, with most selecting healthcare organisations as the interview site. Geographically, the United States contributed four studies, Canada contributed two and China contributed three. The quality assessment revealed that one document received a grade A, while the remaining eight received grade B. All studies met the majority of the criteria outlined in the JBI Quality Assessment Criteria for Qualitative Research. Methodologically, the nine studies employed various qualitative research methods, including descriptive qualitative research, phenomenological research and focus groups, reflecting the diversity of research designs (Table 2). The themes of each study were extracted and are listed in Table 3.

**TABLE 2:** Characteristics of included studies.

Author	Country	Year	Aim	Sample	Methods
Bihari	Canada	2025	To explore the psychological experiences of 13 mothers during their child's transition from paediatric to adult care, focusing on the shift in caregiving roles and the promotion of independence	13	Descriptive qualitative research, semi structured interviews
Li	China	2024	To investigate the caregiving challenges and support needs of family caregivers for adolescents with IBD, focusing on emotional stress and external support systems	16	Phenomenological research, semi structured interviews
Zhou	China	2023	To identify barriers and facilitators in the transition from paediatric to adult care for Chinese adolescents with IBD, emphasising cultural, social and medical factors	13	Phenomenological research, semi structured interviews
Zhang	China	2022	To understand the emotional burden and coping strategies of parents caring for children with crohn's disease, highlighting psychological stress and the need for supportive interventions	11	Phenomenological research, semi structured interviews
Gorbounova	United States	2022	To explore how youth with IBD and their parents perceive and manage pain, and to identify effective coping strategies and the role of healthcare providers in pain management	19	Descriptive qualitative research, semi structured interviews
Easterlin	United States	2020	To examine how paediatric IBD patients and their families adapt to the disease, focusing on coping strategies and the role of social support in managing the challenges	18	Phenomenological research, semi structured interviews
Kluthe	Canada	2018	To explore the immediate reactions of children and parents following a recent IBD diagnosis, focusing on their understanding of the disease and treatment expectations	18	Descriptive qualitative research, semi structured interviews
Gray	United States	2015	To examine the barriers and needs of patients, parents, and healthcare providers during the transition from paediatric to adult care for IBD	16	Focus group
Hommel	United States	2011	To explore factors influencing medication adherence in paediatric IBD patients, including family support, behaviour and organisational tools	16	Descriptive qualitative research, semi structured interviews

**TABLE 3:** Themes of included studies.

Author	Themes
Bihari	5 themes: parental feelings, parents' opinions on child's preparedness, parental involvement in adult care, opinions on adult care, hopes and expectations
Li	15 themes: Distrust, worry, guilt, self‐blame, Economic hardship, disrupted family dynamics, strained family rhythm, knowledge about disease, communication skills, family support, social circles, resistance to being socially labelled, Role adaptation, life re‐prioritisation, behavioural adjustments
Zhou	9 themes: personal characteristics, disease acceptance, transition consciousness, mentality of guilt, self‐efficacy, parent–child separation, transition communication, community support, healthcare environment and policies
Zhang	4 themes: need for medical knowledge, self‐perceived burden, feelings of powerlessness, positive self‐adjustment
Gorbounova	3 themes: IBD threat, fear/worry, biased attending, IBD threat perception
Easterlin	11 themes: treatment decisions, managing social relationships, life transitions, anxiety related to the unknown, managing logistics, managing pain and anxiety with intravenous placement, social support, cognitive strategies for managing emotions, behavioural strategies for managing pain and anxiety, confidence in medical care, life lessons and building resilience
Kluthe	13 themes: changes with diagnosis, feelings upon diagnosis, knowledge of IBD, role of IBD care team, role of child, role of parents, sharing and explaining the diagnosis, understanding and support, adherence, exploring alternatives, looking for information, thoughts on treatment options, continuing on with life
Gray	6 themes: concerns about the adult care, high parent involvement serves, financial concerns, loss of paediatric care relationships, hold adolescents accountable, support for parents
Hommel	3 themes: barriers to treatment adherence, facilitators to treatment adherence, additional factors to treatment adherence

### 3.1 Qualitative Meta‐Synthesis

69 findings were extracted from nine studies and categorised into five themes and 12 subthemes using a pooled integration approach. Detailed information is presented in Table 4.

**TABLE 4:** Overviews of themes and subthemes constructed from the synthesis of the meta.

Theme	Subtheme
Psychological and emotional challenges	Incredulity at diagnosis
	Uncontrollable negative emotions
Heavy caregiving burden	Disrupted life
	High economic costs
Multiple challenges	Social exclusion and stigmatisation
	Adverse effects of medication
	Transition to adult care
	The road ahead
Indispensable external support	Lack of support resources
	Importance of external support
Positive progress	Positive transformation in family life
	Adjusting mindset, maintaining optimism

#### 3.1.1 Theme 1: Psychological and Emotional Challenges

##### 3.1.1.1 Subtheme 1: Incredulity at Diagnosis

The diagnosis of IBD in an adolescent often comes as a shock. Many caregivers report being unprepared for their child's diagnosis, often experiencing emotional reactions such as shock, denial or overwhelming distress (Li et al. 2024). Some caregivers struggled to accept the diagnosis, developing intense negative emotions (Hommel et al. 2011).

I knew my child was diagnosed with inflammatory bowel disease, and I was instantly stunned. It felt like the sky was falling—like fate had been so unfair to her. Now, I don't even want to go to the hospital. I'm scared of doctors discussing her condition; I just can't handle it. (Li et al. 2024)

You know, we all went through a tough time when he was first diagnosed. And it was very scary, and I think we all as a family want to avoid that. (Hommel et al. 2011)

##### 3.1.1.2 Subtheme 2: Uncontrollable Negative Emotions

Throughout caregiving, caregivers often experience self‐blame and guilt due to the discomforts faced by the adolescent (Zhou et al. 2023). Many caregivers question their role in the child's illness, particularly when the condition is erratic, which often leads to feelings of powerlessness and frustration (Zhang and Liu 2022).

I felt very guilty. When I saw other children healthy and my child sick, I felt sorry for him. I just wanted to accompany him and help him as much as I could. (Zhou et al. 2023)

I've heard that this disease is hard to cure. She's so young, will she never be able to get rid of it in her life? (Zhang and Liu 2022)

#### 3.1.2 Theme 2: Heavy Caregiving Burden

##### 3.1.2.1 Subtheme 3: Disrupted Life

Because of adolescents' young age and limited self‐care abilities, caregivers must dedicate more time and energy to their care, negatively impacting their work and personal lives (Li et al. 2024). The adolescent's condition also affects the caregiver's ability to maintain family life. Some caregivers report difficulty balancing caregiving with other family responsibilities (Easterlin et al. 2020). The condition may also disrupt the family's harmonious atmosphere (Easterlin et al. 2020).

Since my child got sick, I've had to take frequent leave to take her to appointments. It's affecting my job, and lately I've been wondering if I should quit and focus entirely on caring for her. (Li et al. 2024)

So it changed our life. It was difficult, even between us (spouses), how to manage the whole family. From my perspective, it was hard, because all the attention was on the girls. I have a little boy, and I had to be fair to make sure he doesn't feel left out. (Easterlin et al. 2020)

It's been challenging. I think when one person in your family has a chronic illness the entire family is affected, there's no way around that. There's still lasting effects. (Easterlin et al. 2020)

##### 3.1.2.2 Subtheme 4: High Economic Costs

Persons living with IBD typically require multidisciplinary treatment, including hormonal therapy, immunosuppressive therapy, biologic therapy and nutritional support (Zhou et al. 2021). The use of biologics, such as infliximab, can be costly, often exceeding thousands of dollars annually, with long‐term use required. Consequently, high treatment costs place significant financial strain on caregivers (Li et al. 2024; Zhang and Liu 2022). Additionally, some caregivers forgo outside employment to better care for their adolescents, further increasing the family's financial burden (Li et al. 2024).

The diagnosis alone cost 30,000 or 40,000 yuan. Annual treatment exceeds 50,000 yuan—we've spent over 200,000 in three years. Now I'm a full‐time caregiver, and it's all on his dad to earn the money. (Zhang and Liu 2022)

I'm really worried about what comes next. The constant hospital visits and high costs are making the financial burden heavier and heavier. (Li et al. 2024)

The financial pressure is huge. A lot of his meds aren't covered by insurance, and I'm not working anymore. We're relying solely on his dad's income. (Li et al. 2024)

#### 3.1.3 Theme 3: Multiple Challenges

##### 3.1.3.1 Subtheme 5: Social Exclusion and Stigmatisation

Adolescents with IBD face numerous challenges, including strict dietary restrictions, frequent abdominal pain and diarrhoea. These issues significantly affect their social activities and can lead to misunderstandings from others. Caregivers are concerned not only with the adolescent's recovery progress but also with their mental health. If the adolescent faces unfair treatment due to the condition, caregivers will seek to protect them (Kluthe et al. 2018).

I would have this need to over‐explain like it was almost my job to make sure everybody understood that what they're saying isn't necessarily accurate… That was upsetting to me too. Some people were dismissing her experience, and other people were so pessimistic about her… (Kluthe et al. 2018)

##### 3.1.3.2 Subtheme 6: Adverse Effects of Medication

The side effects of therapeutic medications are a significant concern for many caregivers. Although these medications are essential for managing the condition, caregivers often worry about their potential adverse effects (Hommel et al. 2011). Many caregivers, in particular, hesitate to use biologics, fearing that their side effects may harm their child's health (Kluthe et al. 2018).

Um, I just know the 6‐MP lowers your immune system, so it also makes it easier for him to catch colds. (Hommel et al. 2011)

We're not against medication, but if possible, we'd prefer to avoid drugs like adalimumab. She's only 14. Maybe when she's older, we'd consider it. (Kluthe et al. 2018)

##### 3.1.3.3 Subtheme 7: Transition to Adult Care

Transitional care is a planned process that transitions adolescents with chronic illnesses from a child‐centered to an adult‐oriented health system. This period is recognised as critical (Tan and Ong 2020). Caregivers often have mixed feelings about transitioning adolescents to adult care systems, ranging from strong support to outright denial (Bihari et al. 2025; Zhou et al. 2023). Most caregivers lack experience with transitioning to adult care systems and thus seek information and suggestions for improving the adult care process (Gray et al. 2015).

At the time of the initial diagnosis, he was quite sad, probably because I spoke too much about the transition and spoke too early. Later I knew that he had to accept his disease before we could move forward. (Zhou et al. 2023)

Where [the pediatric system] will check on you. They will call and do follow up calls where [adult care] it is not that. (Bihari et al. 2025)

I know it's important to let go and let my child be independent. I just need someone to tell me how—what exactly should I do to help my son become more independent? (Gray et al. 2015)

I think teaching kids how to manage their health should be part of every treatment. My daughter doesn't really understand her condition, and I wish the medical staff could educate her directly. (Gray et al. 2015)

##### 3.1.3.4 Subtheme 8: The Road Ahead

IBD treatment is often lengthy and may be lifelong. Caregivers must consider both the adolescent's current condition and plan for their future. Regarding the future of adolescents, some caregivers reported feeling confused (Gorbounova et al. 2022), while others expressed concern and reluctance (Easterlin et al. 2020; Gorbounova et al. 2022). Many caregivers, on the other hand, chose to approach it positively (Easterlin et al. 2020).

How much damage? Will you be able to recover? What is your life going to be like going forward with everything, college, and just normal social stuff and all that? (Gorbounova et al. 2022)

I worry about her a lot. And she's getting ready to go to college this next fall, and it terrifies me that she's going to be there, and I'm not going to be there. (Gorbounova et al. 2022)

But that's something else I worry about too, is as he gets older, are the teachers gonna judge him? Are they gonna hold things against him. (Easterlin et al. 2020)

Now, I need to plan how to support my child through her entire life—college, marriage, kids. I'll always be there for her. (Easterlin et al. 2020)

#### 3.1.4 Theme 4: Indispensable External Support

##### 3.1.4.1 Subtheme 9: Lack of Support Resources

Caregivers of adolescents with IBD face a lack of support, especially authoritative and scientific information about the disease. As a result, many caregivers turn to online platforms for information. However, the variable quality of online information can lead to misinterpretation and unnecessary anxiety (Li et al. 2024; Zhang and Liu 2022). Additionally, the high cost of the disease forces caregivers to seek external support, but the outcomes often fail to meet their needs, leaving their anxiety unresolved (Easterlin et al. 2020).

I'd never heard of this disease before. After the diagnosis, we looked it up online and saw it called an “incurable disease” or “a cancer that doesn't kill.” It terrified me. (Li et al. 2024)

I've read so much online, but my biggest fear is that my child won't grow taller. (Zhang and Liu 2022)

The insurance, they're hard to deal with… They ask for so much, they ask for everything. (Easterlin et al. 2020)

##### 3.1.4.2 Subtheme 10: Importance of External Support

Although caregivers face limited support, external sources of assistance remain a key motivation for their continued caregiving. Support from family and close friends plays a crucial emotional role in this process. Encouragement and assistance from family members help caregivers stay strong and persevere through the challenges of the treatment process (Li et al. 2024). Professional support from healthcare providers boosts caregivers' confidence and sense of reliance. Establishing a stable, trusting relationship with healthcare professionals helps caregivers cope with challenges in the caregiving process (Easterlin et al. 2020).

My husband always tells me, “No matter what happens in the future, as long as we're together as a family, there's nothing to fear.” (Li et al. 2024)

The grandparents came to help us care for the child. Seeing the family support each other gives me faith that these hardships will end someday. (Li et al. 2024)

Some suggested home IV treatments, but I refused. I want to stay at the hospital with the doctors and nurses—they give me confidence and courage. I'm grateful for their support. (Easterlin et al. 2020)

They hand hold me just as much as they hand hold him, so I feel totally supported through it. I feel like they're here to take care of me too. And they do. They have a nice support system here. I can ask questions, they can give me a hug if I needed it. (Easterlin et al. 2020)

#### 3.1.5 Theme 5: Positive Progress

##### 3.1.5.1 Subtheme 11: Positive Transformation in Family Life

During the treatment of adolescents with IBD, many caregivers and family members demonstrated adaptability and resilience. The solidarity and mutual support within the family helped them overcome life's difficulties and challenges. Caregivers proactively learned about the disease and helped adolescents manage their condition (Li et al. 2024). Meanwhile, the adolescents, inspired by the positive family atmosphere, began to improve themselves (Zhou et al. 2023). By learning about disease management and adopting healthier lifestyles, many families are gradually forming better habits (Li et al. 2024).

I search for articles, learn about the disease, and share them with him. I want him to know how to eat, adjust his habits, understand his condition, and protect himself—to live better. (Li et al. 2024)

She took the initiative to communicate with her patient peers on how to manage her disease. Now the disease is all managed by her, and she has changed a lot. (Zhou et al. 2023)

The biggest change is that our family no longer eats fried food. Now, we control the use of oil very well and eat much healthier. Our lifestyle has been adjusted. (Li et al. 2024)

##### 3.1.5.2 Subtheme 12: Adjusting Mindset, Maintaining Optimism

As caregivers gained experience and deeper knowledge of the disease, some experienced a positive shift in their mindset. They began to openly accept the reality, hoping only for their children's health and happiness in the future (Bihari et al. 2025; Li et al. 2024). Caregivers gradually learn to release their burdens and approach life's challenges with a healthier mindset (Easterlin et al. 2020).

He has a stable job, and as long as he can provide for himself, whether he gets married or has children in the future doesn't seem important to me. As long as he is healthy and happy, that's enough. (Li et al. 2024)

I just wanted my child to have as much of a regular life as she can. (Bihari et al. 2025)

Just trying to be very positive and know that a lot of people deal with a lot of challenges in life and this is just what it is, and we're gonna get through it, and you know, trying to just be optimistic. (Easterlin et al. 2020)

Think about the positive part and having the support of each other, I guess that was how we made it so far. (Easterlin et al. 2020)

## 4 Discussion

This study found that most caregivers of adolescents with IBD experienced significant emotional distress, ranging from disbelief at the diagnosis to self‐blame, anxiety and powerlessness during caregiving. The convergence of these negative emotions led to emotional exhaustion, consistent with findings from previous studies (Feingold et al. 2021; Liu et al. 2018; Thapwong et al. 2024). Therefore, measures should be taken to alleviate the psychological burden on caregivers. Nurses working in IBD settings should focus on caregivers' emotional changes, using active listening, empathic dialogue and disease education to reduce psychological stress. Individualised support programs should be implemented for specific psychological issues if needed. Studies have shown that Internet‐based cognitive behavioural therapy effectively alleviates caregiver anxiety, depression and other negative emotions (Biliunaite et al. 2021). Medical institutions and social service agencies can leverage mobile devices to provide psychological interventions for adolescent caregivers with IBD, fostering positive and optimistic emotions.

The caregiving burden on adolescent caregivers with IBD is concerning. This study found that long hours of complex caregiving disrupt the caregiver's daily life, affecting family harmony, as shown in previous research (Banerjee et al. 2024; Pasek et al. 2024; Shattnawi et al. 2023). The heavy caregiving burden negatively impacts both the physical and mental health of caregivers and the quality of care they provide to persons living with IBD (Basilious et al. 2022; Suksatan et al. 2022). An integrated hospital‐community‐family service system is recommended. In this system, the hospital provides medical guidance and multidisciplinary support, the community offers care resources and facilitates communication between the hospital and family, and family care roles are divided to prevent overloading a single caregiver. This system offers personalised, continuous medical services for adolescents, effectively reducing caregivers' burdens (Gao et al. 2022; Wu et al. 2023). The loss of productivity and economic burden on caregivers should also be considered, as highlighted in several studies (Klomberg et al. 2022; Velasco Rodríguez‐Belvís et al. 2024). For diseases like IBD that require long‐term treatment and can be managed on an outpatient basis during remission, healthcare insurance should develop policies to include IBD in the outpatient special disease category, thus reducing caregivers' expenses (Zhou et al. 2021). Enterprises should be encouraged to offer caregivers flexible working hours and telecommuting options to reduce career interruptions caused by frequent leave.

This study found that adolescents with IBD and their caregivers face numerous challenges, including social stigma, medication side effects, limited support, difficult transitions and concerns about the future. These challenges negatively affect the physical and mental health of both adolescents and caregivers, requiring effective measures to address them. Numerous studies highlight the importance of strong social support for caregivers (Hu et al. 2025; Yu et al. 2025; Zou et al. 2024). First, basic knowledge about IBD should be disseminated in schools and communities to raise awareness of the special needs of adolescents with IBD and reduce prejudice and discrimination (Li et al. 2024). Second, IBD‐related foundations and charitable organisations should collaborate to establish a communication platform for families of adolescents with IBD, enabling caregivers to address care‐related challenges in a timely manner. Successful transition to the adult care system is equally important for adolescents with IBD. Caregiver behaviours, such as attachment to the paediatric care setting, underestimating the adolescent's abilities and over‐ or under‐involvement, can hinder this transition (Kumagai et al. 2021). Research has shown that collaboration among caregivers, adolescents and healthcare professionals can facilitate the transition to the adult care system (MacNeill et al. 2022). Therefore, caregivers and adolescents should actively collaborate with physicians and nurses to implement a transition plan that ensures all medical records are successfully transferred to providers in the adult care system.

This study found that caregivers of adolescents with IBD also experience positive outcomes, such as becoming closer to their children, adopting healthier family habits and gaining confidence and hope for their children's future. These positive experiences are referred to as a sense of benefit finding (Liu et al. 2023), which helps caregivers adapt to their roles, reduce negative emotions, alleviate caregiving stress and improve both physical and mental health, thereby enhancing quality of life (Prikken et al. 2022). Studies have shown that self‐efficacy and psychological resilience are key factors contributing to a sense of illness benefit (Zhu et al. 2022). Nurses working in IBD settings can enhance self‐efficacy and psychological resilience by helping caregivers recall positive life events, encouraging hobbies and setting achievable short‐term goals to improve their sense of benefit finding.

### 4.1 Limitations

This study has several limitations. First, the included literature is limited to studies from the United States, Canada and China, restricting geographic and cultural diversity. This limitation may affect the generalizability of the findings across different cultures. Second, quality assessment indicated that only one of the nine included studies was rated as Grade A, while the remaining eight were Grade B. The predominance of moderate‐quality studies may affect the rigour and generalizability of the results. Additionally, Grade B studies may have limitations in study design, data collection or analysis, potentially reducing the reliability of the synthesised findings. Therefore, the findings should be interpreted with caution in light of the available evidence.

## 5 Conclusion

Caregivers of adolescents with IBD face numerous challenges in their caregiving experience. This study identifies several barriers that caregivers encounter, including significant psychological challenges, heavy caregiving burden and the strain on family and social relationships. Despite these obstacles, caregivers exhibited remarkable resilience, adapting over time and finding meaning in their roles. To improve the quality of life for both caregivers and adolescents, healthcare professionals should prioritise individualised, comprehensive support that addresses caregivers' emotional, psychological and practical needs. Moreover, creating a supportive network involving hospitals, communities and families can help alleviate caregivers' burden. Finally, implementing policies to reduce financial burdens and offer flexible care options will further support caregivers and help both them and adolescents towards a healthier future.

## Conflicts of Interest

The authors declare no conflicts of interest.

## Supporting information


**Data S1.** The ENTREQ checklist.

**Data S2.** Search strategy.

## Data Availability

The authors have nothing to report.
