# Supplementary material for: Caregiving Experiences of Caregivers of Adolescents With Inflammatory Bowel Disease: A Qualitative Meta‐Synthesis
Source: Nurs Open. 2025 Jun 27;12(7):e70267. doi: 10.1002/nop2.70267 (PMC12204846; doi:10.1002/nop2.70267)
Supplement: Supplementary file 2 — Data S2. Search strategy. [file NOP2-12-e70267-s002.docx]

Search Strategy

| **Databases** | **Search No.** | **Query** | **Results** |
| --- | --- | --- | --- |
| **Pubmed** | #1 | "adolescent"[MeSH Terms] OR "teen*"[Title/Abstract] OR "youth*"[Title/Abstract] OR "child*"[Title/Abstract] OR "juvenile*"[Title/Abstract] | 3695434 |
|  | #2 | "parents"[MeSH Terms] OR "family member*"[Title/Abstract] OR "care*"[Title/Abstract] OR "relative*"[Title/Abstract] OR "guardian*"[Title/Abstract] | 4244084 |
|  | #3 | "inflammatory bowel diseases"[MeSH Terms] OR "colitis, ulcerative"[MeSH Terms] OR "crohn disease"[MeSH Terms] OR "ulcerative colitis"[Title/Abstract] | 118663 |
|  | #4 | "qualitative research"[MeSH Terms] OR "grounded theory"[MeSH Terms] OR "focus groups"[MeSH Terms] OR "qualitative study"[Title/Abstract] OR "phenomenon"[Title/Abstract] OR "ethnography"[Title/Abstract] OR "narrative"[Title/Abstract] OR "thematic analysis"[Title/Abstract] OR "experience*"[Title/Abstract] OR "feeling*"[Title/Abstract] OR "interview*"[Title/Abstract] | 2299303 |
|  | #5 | #1 AND #2 AND #3 AND #4 | 448 |
| **Embase** | #1 | 'child'/exp OR 'adolescent'/exp OR 'juvenile'/exp OR teen*:ti,ab,kw OR youth*:ti,ab,kw | 4735500 |
|  | #2 | 'inflammatory bowel diseases'/exp OR 'crohn disease'/exp OR 'ulcerative colitis'/exp OR 'colitis, ulcerative':ti,ab,kw | 230923 |
|  | #3 | 'parent'/exp OR 'family member'/exp OR 'relative'/exp OR care*:ti,ab,kw OR guardian*:ti,ab,kw | 3640048 |
|  | #4 | 'qualitative research'/exp OR 'grounded theory'/exp OR 'thematic analysis'/exp OR 'focus groups':ti,ab,kw OR 'qualitative study':ti,ab,kw OR 'phenomenon':ti,ab,kw OR 'ethnography':ti,ab,kw OR 'narrative':ti,ab,kw OR 'experience*':ti,ab,kw OR 'feeling*':ti,ab,kw OR 'interview*':ti,ab,kw | 3115977 |
|  | #5 | #1 AND #2 AND #3 AND #4 | 916 |
| **Cochrane Library** | #1 | MeSH descriptor: [Adolescent] explode all trees | 135760 |
|  | #2 | MeSH descriptor: [Child] explode all trees | 81439 |
|  | #3 | (teen* OR youth* OR juvenile*):ti,ab,kw | 16730 |
|  | #4 | #1 OR #2 OR #3 | 188390 |
|  | #5 | (parent* OR family member* OR care* OR relative* OR guardian*):ti,ab,kw | 476639 |
|  | #6 | MeSH descriptor: [Inflammatory Bowel Diseases] explode all trees | 4981 |
|  | #7 | MeSH descriptor: [Colitis, Ulcerative] explode all trees | 2297 |
|  | #8 | MeSH descriptor: [Crohn Disease] explode all trees | 2316 |
|  | #9 | (ulcerative colitis):ti,ab,kw | 7025 |
|  | #10 | #6 OR #7 OR #8 OR #9 | 9447 |
|  | #11 | MeSH descriptor: [Qualitative Research] explode all trees | 2509 |
|  | #12 | MeSH descriptor: [Grounded Theory] explode all trees | 28 |
|  | #13 | MeSH descriptor: [Focus Groups] explode all trees | 1122 |
|  | #14 | (qualitative research OR grounded theory OR focus groups OR qualitative study OR phenomenon OR ethnography OR narrative OR thematic analysis OR experience* OR feeling* OR interview*):ti,ab,kw | 235330 |
|  | #15 | #9 OR #10 OR #11 OR #12 | 235331 |
|  | #16 | #4 AND #5 AND #10 AND #15 | 45 |
| **Web of Science** | #1 | "child*" OR "adolescent*" OR "teen*" OR "youth*" OR "juvenile*" | 5816405 |
|  | #2 | "parent*" OR "family member*" OR "care*" OR "relative*" OR "guardian*" | 9279568 |
|  | #3 | "inflammatory bowel diseases" OR "colitis, ulcerative" OR "crohn disease" OR "ulcerative colitis" | 180435 |
|  | #4 | "qualitative research" OR "grounded theory" OR "focus groups" OR "qualitative study" OR "phenomenon" OR "ethnography" OR "narrative" OR "thematic analysis" OR "experience*" OR "feeling*" OR "interview*" | 4832387 |
|  | #5 | #1 AND #2 AND #3 AND #4 | 1030 |
| **CINAHL** | #1 | MH Adolescence OR MH Child OR TI ("adolescent*" OR "teen*" OR "youth*" OR "juvenile*") OR AB ("adolescent*" OR "teen*" OR "youth*" OR "juvenile*") | 993860 |
|  | #2 | MH Parents OR TI ("family member*" OR "care*" OR "relative*" OR "guardian*") OR AB ("family member*" OR "care*" OR "relative*" OR "guardian*") | 1431824 |
|  | #3 | MH Inflammatory Bowel Diseases OR MH Colitis, Ulcerative OR MH Crohn Disease OR TI ulcerative colitis OR AB ulcerative colitis | 21964 |
|  | #4 | MH ("Qualitative Studies" OR "Grounded Theory" OR "Focus Groups" OR "Thematic Analysis") OR TI ( "qualitative research" OR "phenomenon" OR "ethnography" OR "narrative" OR "experience*" OR "feeling*" OR "interview*" ) OR AB ( "qualitative research" OR "phenomenon" OR "ethnography" OR "narrative" OR "experience*" OR "feeling*" OR "interview*" ) | 894819 |
|  | #5 | #1 AND #2 AND #3 AND #4 | 158 |
| **PsycInfo** | #1 | MJ("adolescent") OR TIAB("child*" OR "teen*" OR "youth*" OR "juvenile*") | 1103392 |
|  | #2 | MJ("parents") OR TIAB("family member*" OR "care*" OR "relative*" OR "guardian*") | 952738 |
|  | #3 | MJ("crohn disease" OR "inflammatory bowel diseases" OR "colitis, ulcerative" OR "ulcerative colitis") | 983 |
|  | #4 | MJ("thematic analysis" OR "grounded theory" OR "focus groups" OR "qualitative research") OR TIAB("qualitative study" OR "phenomenon" OR "ethnography" OR "narrative" OR "experience*" OR "feeling*" OR "interview*") | 1186285 |
|  | #5 | #1 AND #2 AND #3 AND #4 | 35 |

| **Databases** | **Chinese search strategy** | **English Translation** | **Results** |
| --- | --- | --- | --- |
| **CNKI** | 主题:(儿童 + 青少年 + 中学生 + 小学生 + 未成年) AND 主题:(照顾 + 照护 + 照料 + 护理 + 父母 + 监护人 + 家属 + 亲属) AND 主题:(炎症性肠病 + 克罗恩病 + 溃疡性结肠炎) AND 主题:(体验 + 感受 + 需求 + 经历 + 质性研究 + 定性研究 + 现象学 + 焦点小组 + 扎根理论 + 人种学 + 民族志 + 访谈) | Topic: (children + adolescents + middle school students + primary school students + minors)  AND Topic: (caregiving + caregiving care + caring + nursing + parents + guardians + family members + relatives)  AND Topic: (inflammatory bowel disease + Crohn’s disease + ulcerative colitis)  AND Topic: (experience + feelings + needs + lived experience + qualitative research + qualitative study + phenomenology + focus groups + grounded theory + ethnography + ethnological study + interviews) | 11 |
| **Wangfang** | 主题:(儿童 OR 青少年 OR 中学生 OR 小学生 OR 未成年) AND 主题:(照顾 OR 照护 OR 照料 OR 护理 OR 父母 OR 监护人 OR 家属 OR 亲属) AND 主题:(炎症性肠病 OR 克罗恩病 OR 溃疡性结肠炎) AND 主题:(体验 OR 感受 OR 需求 OR 经历 OR 质性研究 OR 定性研究 OR 现象学 OR 焦点小组 OR 扎根理论 OR 人种学 OR 民族志 OR 访谈) | Topic: (children OR adolescents OR middle school students OR primary school students OR minors)  AND Topic: (caregiving OR caregiving care OR caring OR nursing OR parents OR guardians OR family members OR relatives)  AND Topic: (inflammatory bowel disease OR Crohn’s disease OR ulcerative colitis)  AND Topic: (experience OR feelings OR needs OR lived experience OR qualitative research OR qualitative study OR phenomenology OR focus groups OR grounded theory OR ethnography OR ethnological study OR interviews) | 33 |
| **VIP** | 题名或关键词:(儿童 OR 青少年 OR 中学生 OR 小学生 OR 未成年) AND 题名或关键词:(照顾 OR 照护 OR 照料 OR 护理 OR 父母 OR 监护人 OR 家属 OR 亲属) AND 题名或关键词:(炎症性肠病 OR 克罗恩病 OR 溃疡性结肠炎) AND 题名或关键词:(体验 OR 感受 OR 需求 OR 经历 OR 质性研究 OR 定性研究 OR 现象学 OR 焦点小组 OR 扎根理论 OR 人种学 OR 民族志 OR 访谈) | Title or Keyword: (children OR adolescents OR middle school students OR primary school students OR minors)  AND Title or Keyword: (caregiving OR caregiving care OR caring OR nursing OR parents OR guardians OR family members OR relatives)  AND Title or Keyword: (inflammatory bowel disease OR Crohn’s disease OR ulcerative colitis)  AND Title or Keyword: (experience OR feelings OR needs OR lived experience OR qualitative research OR qualitative study OR phenomenology OR focus groups OR grounded theory OR ethnography OR ethnological study OR interviews) | 4 |
